# Centralized Surgical Care Improves Survival in Non-Functional Well-Differentiated Pancreatic Neuroendocrine Tumors

**DOI:** 10.3390/cancers17183030

**Published:** 2025-09-16

**Authors:** Ahmed Alnajar, Amber Collier, Mehmet Akcin, John I. Lew, Tanaz M. Vaghaiwalla

**Affiliations:** 1 DeWitt Daughtry Department of Surgery, University of Miami Health System, Miami, FL 33136, USA; 2 Division of Endocrine Surgery, DeWitt Daughtry Department of Surgery, University of Miami Health System, Miami, FL 33136, USA

**Keywords:** pancreas, survival outcomes, hospital, volume, disparities

## Abstract

**Background:** Non-functional well-differentiated pancreatic neuroendocrine tumors (WD-PanNETs) are complex, heterogeneous malignancies with variable prognosis. Despite guideline recommendations, disparities in access to specialized care may impact survival. This study examines whether treatment facility type, geographic travel distance, and treatment modalities are associated with survival outcomes in patients diagnosed with WD-PanNETs. **Results:** Among 20,174 patients with WD-PanNETs, the median age was 62 years (IQR: 52–70), and 54% were men. The majority were treated at non-academic hospitals (76%), with 2.9% traveling >250 miles for care. Patients treated at non-academic hospitals (24%) had 50% lower 15-year survival rates compared to those treated at academic hospitals (58%) and integrated hospitals (56%) (*p* < 0.001). Patients traveling >250 miles had a 72% 15-year survival rate, compared to 43% for those traveling <12.5 miles (*p* < 0.001). In the context of facility-type and geographic distance, treatment at non-academic hospitals <250 miles was associated with a 21% higher mortality risk (HR 1.21, 95% CI 1.12–1.31, *p* < 0.001), and treatment at low-volume hospitals increased mortality risk by 25% (HR 1.25, 95% CI 1.14–1.37, *p* < 0.001). In contrast, primary tumor resection was associated with a 64% reduction in mortality risk (HR 0.36, 95% CI 0.33–0.38, *p* < 0.001), which remained significant at all disease stages. **Conclusion:** Treatment at academic or high-volume centers and longer travel distances were associated with improved OS in patients with WD-PanNETs. Primary tumor resection remains critical, while systemic therapies were primarily used in later-stage disease. These findings support policies that improve access to centralized, multidisciplinary care.

## Simple Summary

Non-functional well-differentiated pancreatic neuroendocrine tumors (WD-PanNETs) are a heterogenous group of malignancies with variable prognosis, and disparities in access to specialized care may influence survival. In an analysis of 20,174 patients, most were treated at non-academic hospitals, where survival was significantly lower than at academic or integrated centers. Patients traveling longer distances (>250 miles) had improved survival compared to those treated closer to home. Treatment at non-academic or low-volume centers increased mortality risk, whereas primary tumor resection reduced mortality by 64% across all stages. These findings highlight the importance of centralization, multidisciplinary care, and surgical management to improve survival in WD-PanNETs.

## 1. Introduction

Pancreatic neuroendocrine tumors (PanNETs) account for 1–2% of all pancreatic neoplasms [1]. Although relatively indolent compared to other pancreatic malignancies, more than 50% of well-differentiated, non-functional PanNETs (WD-PanNETs) present with advanced disease [2]. These tumors are distinct from functional PanNETs, which are typically diagnosed earlier due to hormone-related symptoms. While Ki-67 is an important biomarker, it is inconsistently reported in national databases and was, therefore, not used. Consequently, multimodal clinical management is often required to optimize long-term survival and mitigate disease progression. Consequently, multimodal clinical management for non-functional WD-PanNETs is often required to optimize long-term survival and mitigate disease progression. Over the past two decades, advances in diagnostic imaging and increased utilization have contributed to a rise in PanNET detection, resulting in a sevenfold increase in incidence in the United States between 2000 and 2020 [1,3]. Management strategies for WD-PanNETs vary widely based on tumor characteristics, disease stage, and clinical context, and there remains no universal consensus in treatment guidelines. While several expert groups support surveillance for tumors smaller than 2 cm, others advocate surgical resection of all small well-differentiated pancreatic neuroendocrine tumors, regardless of size [4,5,6].

Treatment decisions may also be influenced by social determinants of health (SDH), institutional practices, and patient-specific factors. Prior studies have demonstrated that patients treated at high-volume or academic centers tend to experience improved outcomes compared to those treated at community hospitals [1,7,8]. Additionally, geographic barriers and travel distances have emerged as significant predictors of survival in other cancer types, with patients who travel farther for care often exhibiting better outcomes [9,10,11,12,13,14,15]. However, data examining the combined impact of facility type, hospital volume, and travel distance on survival outcomes in patients with WD-PanNETs remain limited. While previous studies have identified socioeconomic disparities in PanNET care [8], the role of geographic access to specialized centers in shaping long-term outcomes has not been fully characterized in this rare tumor type. This study investigates the association between treatment facility type, geographic travel distance, and survival outcomes in patients with non-functional WD-PanNETs, using a large national cohort to address these gaps in understanding.

## 2. Materials and Methods

### 2.1. Study Design and Data Source

This retrospective cohort study utilized data from the National Cancer Database (NCDB), a hospital-based cancer registry jointly managed by the American College of Surgeons Commission on Cancer and the American Cancer Society. The NCDB collects comprehensive cancer data from >1500 Commission on Cancer–accredited hospitals across the United States, capturing approximately 70% of new cancer diagnoses annually. Data for this study were obtained from a de-identified 2022 NCDB participant user file. The study was deemed exempt from institutional review board (IRB) approval by the University of Miami Miller School of Medicine. Given the low event rate and rarity of WD-PanNETs, we prioritized model stability and generalizability over matched subcohorts, which may bias or underpower results.

### 2.2. Study Population

The study cohort included patients with histologically confirmed, Grade 1 and Grade 2 WD-PanNETs between 2004 and 2021. Patients were excluded if they had Grade 3 tumors, neuroendocrine carcinoma (ICD-O-3 histology codes: 8246, 8041, 8154, 8013, 8158, 8244), or missing survival data. This resulted in a final cohort of 20,174 patients.

### 2.3. Variables and Data Collection

Demographic variables collected included patient age, sex, race, insurance type, and the Charlson–Deyo comorbidity index. Tumor-specific variables included tumor size, stage, and the use of definitive resections (using the site-specific codes 30, 35–37, 40–90) and systemic therapies (i.e., chemotherapy, radiotherapy, hormonal therapy, and immunotherapy). Facility-level data were defined using Commission on Cancer designations in the NCDB: Academic/Research Programs have postgraduate training and research infrastructure; Integrated Network Programs are high-volume systems with coordinated care across sites; Community Programs treat 100–500 new cases annually. Community programs within 250 miles were further subclassified as Comprehensive (meeting higher accreditation standards) vs. non-comprehensive. “Other facility types” includes facilities lacking standard designation.

Geographic access was assessed by calculating the distance traveled for treatment, grouped into three categories: 0–12.49 miles, 12.5–249 miles, and >250 miles, as previously established [16]. Hospital volume defined by the number cases diagnosed in each participating center per year during the study period (2004–2021), categorized into tertiles: high-volume centers (>81 cases/year), moderate-volume centers (31–81 cases/year), and low-volume centers (≤30 cases/year).

Overall survival (OS) was defined as time from diagnosis to last follow-up or death, as reported in the NCDB.

### 2.4. Statistical Analysis

Descriptive statistics were used to summarize baseline characteristics. Missing data were handled using mode imputation for categorical variables and mean imputation for continuous variables. All results are based on the imputed data (a Supplementary Table provides details on missing data patterns). Continuous variables were reported as medians with interquartile ranges, while categorical variables were presented as frequencies and percentages. Group comparisons were made using Wilcoxon rank-sum tests for continuous variables and Pearson’s Chi-squared tests for categorical variables.

Kaplan–Meier survival curves were generated to assess OS differences between treatment groups, with the log-rank test used to determine statistical significance. Cox proportional hazards models were applied to identify independent predictors of overall mortality (defined as time from diagnosis to death or last contact, consistent with NCDB survival capture). The multivariable model was adjusted for confounding factors such as hospital type, treatment distance, and tumor resection status, which were selected as a priori based on their clinical relevance, availability in the NCDB, and the previously published literature. Sensitivity analysis, excluding patients diagnosed in 2020–2021 to account for COVID-related treatment delays, was performed. The interaction between facility types and geographic distance was tested and reported, and then the main effect estimates for hospital type and distance in addition to the stratified categories were reported in the final multivariable model. In addition to adjusting for clinically and statistically significant variables, propensity score matching or weighting was not applied. This was due to the study’s primary focus on system-level characteristics and the need to preserve the full sample for rare subgroup analysis. In Cox modeling, the proportional hazards assumption was tested using the Schoenfeld residuals test. Model fit was assessed using concordance statistics and the Akaike Information Criterion (AIC). Variance inflation factors (VIFs) were calculated to assess multicollinearity, with a VIF < 4 used to rule out significant collinearity. All statistical analyses were performed using R version 4.4.2 (Pile of Leaves) with the ‘survminer’ and ‘gtsummary’ packages, along with their dependencies.

## 3. Results

### 3.1. Patient Characteristics

**Figure 1:**
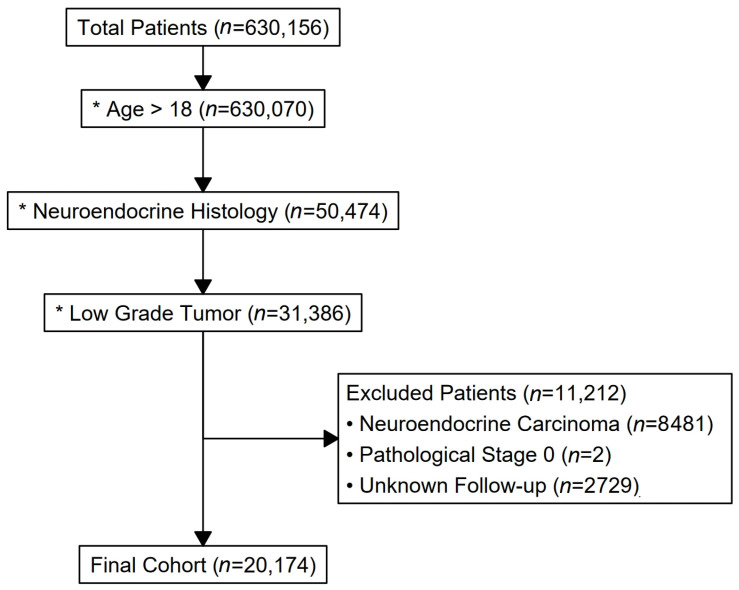
FlowchartPatient Selection for the Study of Well-Differentiated Non-functional Pancreatic Neuroendocrine Tumors.

A total of 20,174 patients with non-functional WD-PanNETs were included in the study (Figure 1). Median age was 62 years (IQR: 52–70), with 54% male and 46% female patients. Most patients (76%) were treated at academic or integrated hospitals, while 24% received care at other facility types. Sixty-three percent of patients traveled 12.5 to 249 miles for treatment, and 2.9% traveled more than 250 miles (Table 1).

Tumor size was similar between non-academic and academic centers (median 24 mm), with a slight but statistically significant difference in IQRs (16–40 mm vs. 15–37 mm, *p* < 0.001).

Patients at non-academic centers received systemic therapies—including chemotherapy, hormonal therapy, and radiotherapy—more frequently than those treated at academic centers. Primary tumor resection was performed in 59% of cases at non-academic hospitals, compared to 68% at academic hospitals (*p* < 0.001) (Supplementary Table S2A). Advanced therapies, such as chemotherapy and radiotherapy, were mainly used for stage IV disease, with 26% and 7% of these patients who received chemotherapy and radiotherapy, respectively, compared to <6% and 1% in stages I–III (*p* < 0.001) (Supplementary Table S2B).

### 3.2. Survival Outcomes by Treatment Setting

**Figure 2:**
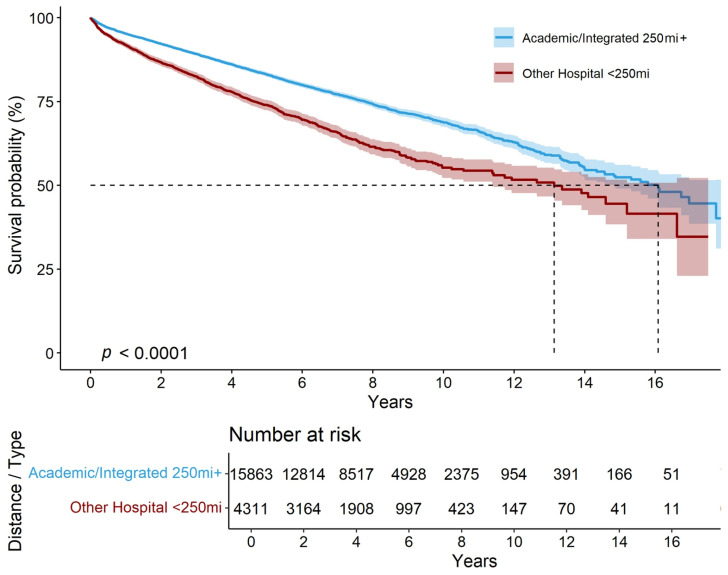
Kaplan–Meier survival curves by geographic distance to treatment facility and facility type.

Kaplan–Meier analysis revealed significant survival differences based on hospital type (Figure 2). Patients treated at non-academic hospitals <250 miles had a 15-year survival rate of 45% (95% CI: 38–52%) and a median survival of 13 years, compared to 52% (95% CI: 49–56%) and 15 years for those treated outside this range (Supplementary Table S3, Figure S2).

Patients at high-volume centers had a median survival of 16 years, compared to 14 years at moderate- and low-volume centers, with survival rates higher at high- and moderate-volume centers over time (Supplementary Figure S1). Patients treated at academic hospitals had the best outcomes, with a 15-year survival rate of 54% (95% CI: 51–58%), followed by integrated hospitals (46%, 95% CI: 38–56%) and other facility types (44%, 95% CI: 38–51%) (*p* < 0.001) (Supplementary Table S3, Supplementary Figure S2).

Additional survival analysis assessing facilities distance demonstrated that patients traveling >250 miles for care had the highest survival rates (60%, 95% CI: 48–76%) and a median survival of 15 years, compared to 48% (95% CI: 43–54%) and 14 years for those living within 12.5 miles of the treatment center (*p* < 0.001) (Supplementary Table S3, Supplementary Figure S3).

### 3.3. Impact of Primary Tumor Resection and Systemic Therapies

Primary tumor resection was associated with significantly improved survival. Patients who underwent resection had a 15-year survival rate of 58% (95% CI: 55–62%) and a median survival of 18 years (95% CI: 16–NE), compared to 30% (95% CI: 24–38%) and 9 years (95% CI: 6.1–9.7) for those who did not undergo surgery.

Patients requiring chemotherapy or radiotherapy had poorer survival. Chemotherapy was associated with a 15-year survival rate of 20% (95% CI: 14–28%) and a median survival of 5.2 years (95% CI: 4.7–5.5), while radiotherapy showed similar outcomes with a 15-year survival rate of 25% (95% CI: 16–40%) and a median survival of 5.6 years (95% CI: 4.5–6.6) (Supplementary Table S3).

### 3.4. Cox Proportional Hazards Analysis

Univariable Cox analysis identified treatment at community hospitals <250 miles as a significant factor for higher mortality (HR: 1.62, 95% CI: 1.51–1.74, *p* < 0.001). Multivariable analysis demonstrated this association with an HR of 1.21 (95% CI: 1.12–1.31, *p* < 0.001) for community hospitals <250 miles. Integrated hospitals were associated with a modest increase in mortality compared to academic centers (HR: 1.24, 95% CI: 1.14–1.36, *p* < 0.001).

Treatment at integrated and academic hospitals >250 miles was linked to the lowest mortality risk (HR: 0.44, 95% CI: 0.34–0.55, *p* < 0.001), while treatment at these hospitals <250 miles still significantly reduced mortality compared to community hospitals (HR: 0.62, 95% CI: 0.58–0.67, *p* < 0.001). Within community hospitals <250 miles, there was no significant survival benefit between comprehensive and non-comprehensive programs (HR: 0.84, 95% CI: 0.68–1.03, *p* = 0.090), although other facility types (including academic/integrated programs) showed a notable survival advantage (HR: 0.52, 95% CI: 0.43–0.64, *p* < 0.001). Regarding geographic distance, traveling >250 miles for treatment was associated with a significantly lower mortality risk compared to living <12.5 miles of the treatment center (HR: 0.52, 95% CI: 0.42–0.65, *p* < 0.001).

Multivariable Cox analysis demonstrated that both primary tumor resection (HR: 0.36, 95% CI: 0.33–0.38, *p* < 0.001) and centralized care were associated with significant survival benefits. Age > 65 years was strongly linked to worse survival (HR: 1.70, 95% CI: 1.57–1.85, *p* < 0.001). Women had a 16% reduced mortality risk compared to men (HR: 0.84, 95% CI: 0.79–0.90, *p* < 0.001). Tumor stage was also a major determinant of survival, with stage IV disease associated with an HR of 1.90 (95% CI: 1.74–2.08, *p* < 0.001) (Table 2).

Stage-specific analyses showed that primary tumor resection significantly reduced mortality across all advanced stages: for stage II, HR: 0.46 (95% CI: 0.26–0.82, *p* = 0.008); for stage III, HR: 0.31 (95% CI: 0.14–0.67, *p* = 0.003); and for stage IV, HR: 0.21 (95% CI: 0.15–0.32, *p* < 0.001) (Supplementary Table S4). A sensitivity analysis excluding the 26% of patients diagnosed in 2020–2021, during the height of the COVID-19 pandemic, showed similar findings and is presented in Supplementary Table S5.

## 4. Discussion

Our findings highlight the survival benefit of high-volume centers and academic institutions, with geographic distance serving as a surrogate for access to such care. These findings underscore the need for referral systems to reduce avoidable disparities. Specifically, treatment at non-academic hospitals within 250 miles was associated with a 21% worse survival compared to care at academic or integrated hospitals within 250 miles or at non-academic hospitals beyond 250 miles. While the adverse effects of structural access barriers on cancer outcomes have been well-documented across multiple cancer types [10,11,12,13,14], limited data exist for PanNETs, which are complex malignancies with heterogenous presentation and prognosis. A prior study by Underwood et al. showed that lower household income and educational attainment were associated with worse survival among patients with PanNETs [8], demonstrating the significant influence of socioeconomic status. Complementing these findings, the present study evaluates geographic access to centralized care and demonstrates that increased travel distance to high-volume or academic centers is independently associated with improved overall survival.

These study findings underscore the critical role of facility type in long-term outcomes for patients with PanNETs. Academic and high-volume centers are known to deliver superior outcomes in pancreatic and other cancers [9,11,12,13,14,15], which may be due to multidisciplinary expertise, access to advanced surgical techniques and novel therapies. Furthermore, there may be greater adherence to evidence-based guidelines, access to clinical trials and comprehensive cancer management, which are factors that collectively improve survival and reduce complications [14,15,17,18]. These results are consistent with those of Patel et al., who reported improved disease-specific survival among patients with non-functional PanNETs treated at high-volume centers (≥5 cases/year; median survival 63 vs. 32 months for locoregional disease; HR 0.63, *p* = 0.002) [7]. Building upon Patel et al.’s single-state analysis, the present study evaluates a national cohort of 20,174 patients and employs multivariable Cox models adjusted for both facility type and travel distance. Using a large, diverse sample with refined exposure definitions, this study demonstrates that treatment at high-volume institutions is independently associated with improved survival. These findings support policies to enhance access to high-volume surgical centers, with strengthened referral systems and expanded patient support initiatives helping to overcome geographic barriers and ensure equitable care [19,20,21,22]. Moreover, identifying high-risk groups—such as elderly patients, those with comorbidities, or larger tumors—may inform more tailored perioperative planning and long-term follow-up strategies in everyday practice.

When examining the role of surgery in the care of PanNets, primary tumor resection remained a key determinant of survival (HR 0.36, 95% CI 0.33–0.38, *p* < 0.001). Patients who underwent surgical resection demonstrated a 15-year survival rate of 58% and a median survival of 18 years, compared to a 15-year survival of 30% and a median survival of 9 years among those who did not undergo surgery. Furthermore, surgical intervention was associated with improved survival across all tumor sizes and stages, aligning with prior studies that examined resection in patients with tumors >1 cm [1,3]. While current recommendations remain controversial regarding the resection of tumors <2 cm due to limited observed benefit and potential surgical risks [1,3,23,24,25,26,27], the present study findings demonstrate that primary tumor resection is associated with a survival advantage across all primary tumor sizes. This is particularly relevant given the heterogeneous behavior of PanNETs and the potential to mitigate recurrence risk even for patients with tumors ≤2 cm [28,29,30,31,32]. Given the role of surgery in the management of PanNETs, evaluating centralization of care, facility factors, and social determinants is particularly relevant for surgical outcomes.

This study has limitations inherent to its retrospective design. The NCDB lacks detailed clinical and molecular data, such as Ki-67 index or somatostatin receptor status, which are important for personalized management. Additionally, recurrence and long-term quality of life data are unavailable, limiting assessment of treatment durability. Findings are generalizable only to patients treated at Commission on Cancer–accredited facilities. This study is also limited by missing data on certain covariates, which were addressed with mean or mode imputation. While facility type and geographic distance serve as proxies for centralized care, further research is needed to elucidate the specific institutional factors responsible for improved outcomes. Selection bias remains a concern, as patients who travel farther or undergo surgery may differ in health status or tumor biology. Following adjustment for age, stage, and comorbidity, both treatment at high-volume centers and longer travel distance remained independently associated with improved overall survival. This suggests a benefit of centralized care beyond measured patient-related factors, while residual confounding cannot be excluded. The absence of granularity for treatment-specific data, including use of peptide receptor radionuclide therapy or other systemic therapies, further limits the analysis of non-surgical interventions.

## 5. Conclusions

In conclusion, treatment at academic hospitals, care at high-volume centers, and greater travel distance were each associated with improved survival in patients with non-functional WD-PanNETs. Primary tumor resection remained a strong predictor of improved survival across all tumor sizes and disease stages. These findings highlight the importance of individualized treatment planning that incorporates both clinical and structural factors, including access to specialized care. Health policy efforts to foster referral pathways to centralized centers and mitigate travel-related barriers may promote equitable, high-quality care for patients with WD-PanNETs.

## Figures and Tables

**Table 1:** Demographic and clinical characteristics of patients with well-differentiated non-functional pancreatic neuroendocrine tumors.

Characteristic	Overall, *n* = 20,174 ^1^	Non-Academic Hospitals, *n* = 4908 ^1^	Academic/Integrated Hospitals, *n* = 15,266 ^1^	*p*-Value ^2^
Age at Diagnosis	62 (52, 70)	65 (57, 73)	61 (51, 70)	<0.001
Age > 65 Years	8730 (43%)	2585 (53%)	6145 (40%)	<0.001
Sex				0.12
Female	9272 (46%)	2192 (45%)	7080 (46%)	
Male	10,902 (54%)	2716 (55%)	8186 (54%)	
Private Insurance	9409 (47%)	1987 (40%)	7422 (49%)	<0.001
Hospital Distance (mi)				<0.001
0 to 12.49	6966 (35%)	2251 (46%)	4715 (31%)	
12.5 to 249	12,624 (63%)	2617 (53%)	10,007 (66%)	
250+	584 (2.9%)	40 (0.8%)	544 (3.6%)	
Hospital Volume (tertiles)				<0.001
High	10,841 (54%)	1088 (22%)	9753 (64%)	
Moderate	6623 (33%)	2257 (46%)	4366 (29%)	
Low	2710 (13%)	1563 (32%)	1147 (7.5%)	
Charlson/Deyo Score				**<0.001**
0	14,024 (70%)	3247 (66%)	10,777 (71%)	
1	4036 (20%)	1106 (23%)	2930 (19%)	
2	1214 (6.0%)	318 (6.5%)	896 (5.9%)	
3	900 (4.5%)	237 (4.8%)	663 (4.3%)	
Pathological Stage				<0.001
Stage I	12,962 (64%)	3258 (66%)	9704 (64%)	
Stage II	4305 (21%)	943 (19%)	3362 (22%)	
Stage III	1171 (5.8%)	256 (5.2%)	915 (6.0%)	
Stage IV	1736 (8.6%)	451 (9.2%)	1285 (8.4%)	
Tumor Size (mm)	24 (15, 38)	24 (16, 40)	24 (15, 37)	<0.001

^1^ Median (IQR); n (%), ^2^ Wilcoxon rank sum test; Pearson’s Chi-squared test.

**Table 2:** Univariable and multivariable Cox analysis for hospital distance, facility type, and demographics.

	Univariable Module			Multivariable Module		
Characteristic	HR ^1^	95% CI ^1^	*p*-Value	HR ^1^	95% CI ^1^	*p*-Value
Hospital Distance (mi)						
0 to 12.49	—	—				
12.5 to 249	0.85	0.80, 0.91	<0.001			
250+	0.52	0.42, 0.65	<0.001			
Facility Type						
Academic	—	—				
Integrated	1.24	1.14, 1.36	<0.001			
Community	1.76	1.64, 1.89	<0.001			
Non-Community Hospital Distance						
Community (within 250 mi)	—	—				
Integrated and Academic (beyond 250 mi)	0.44	0.34, 0.55	<0.001			
Integrated and Academic (within 250 mi)	0.62	0.58, 0.67	<0.001			
Community Hospital Types within 250 mi						
Community (within 250 mi)	—	—				
Comprehensive Community (within 250 mi)	0.84	0.68, 1.03	0.090			
Other facility types	0.52	0.43, 0.64	<0.001			
Non-Academic Hospitals < 250 mi	1.62	1.51, 1.74	<0.001	1.21	1.12, 1.31	<0.001
Age > 65 Years	2.34	2.19, 2.49	<0.001	1.70	1.57, 1.85	<0.001
Female Sex	0.77	0.73, 0.83	<0.001	0.84	0.79, 0.90	<0.001
African American (Ref: White)	1.01	0.92, 1.11	0.9	1.01	0.92, 1.12	0.8
Private Insurance	0.45	0.42, 0.48	<0.001	0.70	0.65, 0.76	<0.001
Charlson/Deyo Score						
0	—	—		—	—	
1	1.23	1.14, 1.32	<0.001	1.18	1.09, 1.27	<0.001
2	1.58	1.41, 1.78	<0.001	1.42	1.26, 1.60	<0.001
3	2.34	2.07, 2.65	<0.001	1.93	1.71, 2.19	<0.001
Tumor Size						
<1 cm	—	—		—	—	
1–1.5 cm	0.84	0.71, 0.98	0.026	0.81	0.69, 0.95	0.009
1.6–2 cm	0.96	0.81, 1.13	0.6	0.99	0.84, 1.17	>0.9
>2 cm	1.80	1.57, 2.06	<0.001	1.71	1.49, 1.97	<0.001
Tumor Grade						
G1	—	—		—	—	
G2	1.33	1.24, 1.43	<0.001	1.24	1.15, 1.33	<0.001
Pathological Stage						
Stage I	—	—		—	—	
Stage II	0.67	0.61, 0.74	<0.001	0.88	0.79, 0.97	0.012
Stage III	0.75	0.62, 0.90	0.002	0.98	0.81, 1.19	0.9
Stage IV	2.41	2.21, 2.62	<0.001	1.90	1.74, 2.08	<0.001
Hospital Volume (tertiles)						
High	—	—		—	—	
Moderate	1.34	1.25, 1.43	<0.001	1.20	1.12, 1.29	<0.001
Low	1.81	1.66, 1.97	<0.001	1.25	1.14, 1.37	<0.001
Primary Tumor Resection	0.31	0.29, 0.33	<0.001	0.36	0.33, 0.38	<0.001

^1^ HR = Hazard Ratio, CI = Confidence Interval.

## Supplementary Materials

The following supporting information can be downloaded at: https://www.mdpi.com/article/10.3390/cancers17183030/s1, **Table S1.**: Detailed Summaries of Missing Data Patterns in Demographic and Clinical Characteristics of Patients with WD-PanNETs. **Table S2.** A: Comparison of Treatment Modalities and Outcomes in Non-Academic vs. Academic/Integrated Hospitals. **Table S2.** B: Treatment Modalities and Outcomes Across Pathological Stages. **Table S3.** Five-Year and Fifteen-Year Survival Rates and Median Survival by Demographics and Treatment Factors. **Table S4.** Interaction of Stage and Primary Tumor Resection on Survival Outcomes. **Table S5.** Sensitivity analysis for COVID effect on Factors Associated with Mortality. **Figure S1.** Kaplan-Meier Survival Curves by Hospital Volume. **Figure S2.** Kaplan-Meier Survival Curves by Facility Type. **Figure S3.** Kaplan-Meier Survival Curves by Geographic Distance to Treatment Facility.
