# Supplementary material for: Centralized Surgical Care Improves Survival in Non-Functional Well-Differentiated Pancreatic Neuroendocrine Tumors
Source: Cancers (Basel). 2025 Sep 16;17(18):3030. doi: 10.3390/cancers17183030 (PMC12468637; doi:10.3390/cancers17183030)
Supplement: Supplementary file 1 [file cancers-17-03030-s001.zip › cancers-3844308-supplementary.pdf]

# Supplementary

**Table S1.** : Detailed Summaries of Missing Data Patterns in Demographic and Clinical Characteristics of Patients with WD-PanNETs.

| Characteristic | *n* = 20,174[1] |
|---|---|
| Age at Diagnosis | 62 (52, 70) |
| Age > 65 Years | 8,730 (43%) |
| Sex | |
| Female | 9,272 (46%) |
| Male | 10,902 (54%) |
| Private Insurance | 9,409 (47%) |
| Unknown | 317 |
| Hospital Distance (mi) | |
| 0 to 12.49 | 6,966 (40%) |
| 12.5 to 249 | 9,772 (56%) |
| 250+ | 584 (3.4%) |
| Unknown | 2,852 |
| Hospital Volume (tertiles) | |
| High | 10,841 (54%) |
| Moderate | 6,623 (33%) |
| Low | 2,710 (13%) |
| Charlson/Deyo Score | |
| 0 | 14,024 (70%) |
| 1 | 4,036 (20%) |
| 2 | 1,214 (6.0%) |
| 3 | 900 (4.5%) |
| Pathological Stage | |
| Stage I | 5,919 (45%) |
| Stage II | 4,305 (33%) |
| Stage III | 1,171 (8.9%) |
| Stage IV | 1,736 (13%) |
| Unknown | 7,043 |
| Facility Type | |
| Academic | 10,388 (56%) |
| Integrated | 3,346 (18%) |
| Community | 4,908 (26%) |
| Unknown | 1,532 |

[1]Median (Q1, Q3); n (%).

**Table S2.** A: Comparison of Treatment Modalities and Outcomes in Non-Academic vs. Academic/Integrated Hospitals.

| Characteristic | Non-academic Hospitals, *n* = 4,908[1] | Academic/Integrated Hospitals, *n* = 15,266[1] | *p*-value[2] |
|---|---|---|---|
| Primary Tumor Resection | 2,882 (59%) | 10,421 (68%) | **< 0.001** |
| Chemotherapy | 365 (7.4%) | 969 (6.3%) | **0.008** |
| Radiotherapy | 117 (2.4%) | 256 (1.7%) | **0.001** |
| Hormonal Therapy | 440 (9.0%) | 926 (6.1%) | **< 0.001** |
| Immunotherapy | 18 (0.4%) | 93 (0.6%) | **0.046** |

[1]n (%)

[2]Pearson's Chi-squared test.

**Table S2.** B: Treatment Modalities and Outcomes Across Pathological Stages.

| **Characteristic** | **Stage I** $n$ = 12,962[1] | **Stage II** $n$ = 4,305[1] | **Stage III** $n$ = 1,171[1] | **Stage IV** $n$ = 1,736[1] | $p$**-value**[2] |
|---|---|---|---|---|---|
| Primary Tumor Resection | 7,100 (55%) | 4,205 (98%) | 1,132 (97%) | 866 (50%) | |
| Chemotherapy | 770 (5.9%) | 81 (1.9%) | 32 (2.7%) | 451 (26%) | **< 0.001** |
| Radiotherapy | 201 (1.6%) | 39 (0.9%) | 14 (1.2%) | 119 (6.9%) | **< 0.001** |
| Hormonal Therapy | 760 (5.9%) | 46 (1.1%) | 38 (3.2%) | 522 (30%) | **< 0.001** |
| Immunotherapy | 77 (0.6%) | 7 (0.2%) | 1 (<0.1%) | 26 (1.5%) | **< 0.001** |
| Facility Type | | | | | **< 0.001** |
| Academic | 7,537 (58%) | 2,636 (61%) | 724 (62%) | 1,023 (59%) | |
| Integrated | 2,167 (17%) | 726 (17%) | 191 (16%) | 262 (15%) | |
| Non-academic | 3,258 (25%) | 943 (22%) | 256 (22%) | 451 (26%) | |

[1]n (%)

[2]Fisher's exact test; Pearson's Chi-squared test.

**Table S3.** Five-Year and Fifteen-Year Survival Rates and Median Survival by Demographics and Treatment Factors.

| **Characteristic** | **5 Year** | **15 Year** | **Median Survival** |
|---|---|---|---|
| Overall | 81% (81%, 82%) | 51% (48%, 54%) | 15 (14, 17) |
| Age > 65 Years | | | |
| No | 87% (86%, 88%) | 60% (56%, 65%) | 19 (17, —) |
| Yes | 73% (72%, 74%) | 34% (30%, 40%) | 10 (9.8, 11) |
| Community Hospitals < 250 mi | | | |
| No | 83% (82%, 84%) | 52% (49%, 56%) | 16 (15, —) |
| Yes | 74% (72%, 76%) | 45% (38%, 52%) | 13 (11, —) |
| Community Hospital Types within 250 mi | | | |
| Community (within 250 mi) | 69% (63%, 75%) | 41% (26%, 64%) | 10 (7.9, —) |
| Comprehensive Community (within 250 mi) | 74% (73%, 76%) | 45% (38%, 52%) | 13 (11, —) |
| Other | 83% (82%, 84%) | 52% (49%, 56%) | 16 (15, —) |
| Non-Community Hospital Distance | | | |
| Community (within 250 mi) | 74% (72%, 76%) | 45% (38%, 52%) | 13 (11, —) |
| Integrated & Academic (beyond 250 mi) | 87% (84%, 91%) | 56% (41%, 75%) | 15 (15, —) |
| Integrated & Academic (within 250 mi) | 83% (82%, 84%) | 52% (49%, 56%) | 16 (15, —) |
| Facility Type | | | |
| Academic | 84% (83%, 85%) | 54% (51%, 58%) | 17 (15, —) |
| Integrated | 81% (79%, 82%) | 46% (38%, 56%) | 14 (13, —) |
| Community | 74% (72%, 75%) | 44% (38%, 51%) | 13 (11, 15) |
| Hospital Distance (mi) | | | |
| 0 to 12.49 | 79% (78%, 80%) | 48% (43%, 54%) | 15 (14, —) |
| 12.5 to 249 | 82% (81%, 83%) | 52% (48%, 56%) | 17 (14, —) |
| 250+ | 88% (85%, 91%) | 60% (48%, 76%) | 18 (15, —) |
| Primary Tumor Resection | | | |
| No | 65% (64%, 67%) | 30% (24%, 38%) | 9.0 (8.3, 9.7) |
| Yes | 88% (88%, 89%) | 58% (55%, 62%) | 18 (16, —) |
| Chemotherapy | | | |
| No | 83% (83%, 84%) | 54% (50%, 57%) | 17 (15, —) |
| Yes | 52% (49%, 55%) | 20% (14%, 28%) | 5.2 (4.7, 5.5) |
| Radiotherapy | | | |
| No | 82% (81%, 82%) | 51% (48%, 55%) | 16 (15, 17) |
| Yes | 52% (47%, 59%) | 25% (16%, 40%) | 5.6 (4.5, 6.6) |
| Hormonal Therapy | | | |
| No | 83% (82%, 83%) | 52% (49%, 56%) | 16 (15, —) |
| Yes | 56% (53%, 60%) | — (—, —) | 6.0 (5.4, 6.7) |

| Characteristic | 5 Year | 15 Year | Median Survival |
|---|---|---|---|
| Pathological Stage | | | |
| Stage I | 81% (81%, 82%) | 52% (49%, 56%) | 16 (15, —) |
| Stage II | 88% (87%, 89%) | 55% (47%, 64%) | 17 (14, —) |
| Stage III | 85% (82%, 88%) | 46% (28%, 76%) | 9.7 (7.8, —) |
| Stage IV | 60% (58%, 63%) | 23% (14%, 39%) | 6.9 (6.4, 7.6) |
| Hospital Volume (tertiles) | | | |
| High | 84% (83%, 85%) | 54% (50%, 58%) | 16 (15, —) |
| Moderate | 80% (79%, 81%) | 49% (43%, 55%) | 14 (13, —) |
| Low | 73% (71%, 75%) | 44% (37%, 51%) | 14 (12, 16) |

**Table S4.** Interaction of Stage and Primary Tumor Resection on Survival Outcomes.

| Characteristic | HR[1] | 95% CI[1] | p-value |
|---|---|---|---|
| Pathological Stage | | | |
| Stage I | — | — | |
| Stage II | 2.86 | 1.62, 5.02 | **<0.001** |
| Stage III | 5.06 | 2.42, 10.6 | **<0.001** |
| Stage IV | 12.1 | 8.51, 17.3 | **<0.001** |
| Primary Tumor Resection | 1.43 | 1.01, 2.02 | **0.047** |
| Pathological Stage * Primary Tumor Resection | | | |
| Stage II * Primary Tumor Resection | 0.46 | 0.26, 0.82 | **0.008** |
| Stage III * Primary Tumor Resection | 0.31 | 0.14, 0.67 | **0.003** |
| Stage IV * Primary Tumor Resection | 0.21 | 0.15, 0.32 | **<0.001** |

[1]HR = Hazard Ratio, CI = Confidence Interval.

**Table S5.** Sensitivity analysis for COVID effect on Factors Associated with Mortality.

| | Original module | | | Before COVID-19 model | | |
|---|---|---|---|---|---|---|
| Characteristic | HR | 95% CI | p-value | HR | 95% CI | p-value |
| Community Hospitals <250mi | 1.21 | 1.12, 1.31 | **<0.001** | 1.23 | 1.13, 1.33 | **<0.001** |
| Age>65 Years | 1.70 | 1.57, 1.85 | **<0.001** | 1.73 | 1.59, 1.89 | **<0.001** |
| Female Sex | 0.84 | 0.79, 0.90 | **<0.001** | 0.85 | 0.79, 0.91 | **<0.001** |
| African American (Ref: White) | 1.01 | 0.92, 1.12 | 0.8 | 0.96 | 0.86, 1.07 | 0.5 |
| Private Insurance | 0.70 | 0.65, 0.76 | **<0.001** | 0.69 | 0.63, 0.75 | **<0.001** |
| Charlson/Deyo Score | | | | | | |
| 0 | — | — | | — | — | |
| 1 | 1.18 | 1.09, 1.27 | **<0.001** | 1.20 | 1.10, 1.30 | **<0.001** |
| 2 | 1.42 | 1.26, 1.60 | **<0.001** | 1.45 | 1.27, 1.65 | **<0.001** |
| 3 | 1.93 | 1.71, 2.19 | **<0.001** | 1.99 | 1.73, 2.29 | **<0.001** |
| Tumor Size | | | | | | |
| <1 cm | — | — | | — | — | |
| 1-1.5 cm | 0.81 | 0.69, 0.95 | **0.009** | 0.87 | 0.73, 1.04 | 0.13 |
| 1.6-2 cm | 0.99 | 0.84, 1.17 | >0.9 | 1.05 | 0.87, 1.26 | 0.6 |
| >2 cm | 1.71 | 1.49, 1.97 | **<0.001** | 1.78 | 1.52, 2.08 | **<0.001** |
| Tumor Grade | | | | | | |
| G1 | — | — | | — | — | |
| G2 | 1.24 | 1.15, 1.33 | **<0.001** | 1.22 | 1.13, 1.32 | **<0.001** |
| Pathological Stage | | | | | | |
| Stage I | — | — | | — | — | |
| Stage II | 0.88 | 0.79, 0.97 | **0.012** | 0.93 | 0.84, 1.04 | 0.2 |
| Stage III | 0.98 | 0.81, 1.19 | 0.9 | 1.02 | 0.82, 1.28 | 0.8 |
| Stage IV | 1.90 | 1.74, 2.08 | **<0.001** | 1.91 | 1.74, 2.11 | **<0.001** |
| Hospital Volume (tertiles) | | | | | | |
| High | — | — | | — | — | |
| Moderate | 1.20 | 1.12, 1.29 | **<0.001** | 1.19 | 1.10, 1.29 | **<0.001** |
| Low | 1.25 | 1.14, 1.37 | **<0.001** | 1.26 | 1.14, 1.39 | **<0.001** |
| Primary Tumor Resection | 0.36 | 0.33, 0.38 | **<0.001** | 0.36 | 0.33, 0.39 | **<0.001** |

Abbreviations: CI = Confidence Interval, HR = Hazard Ratio.

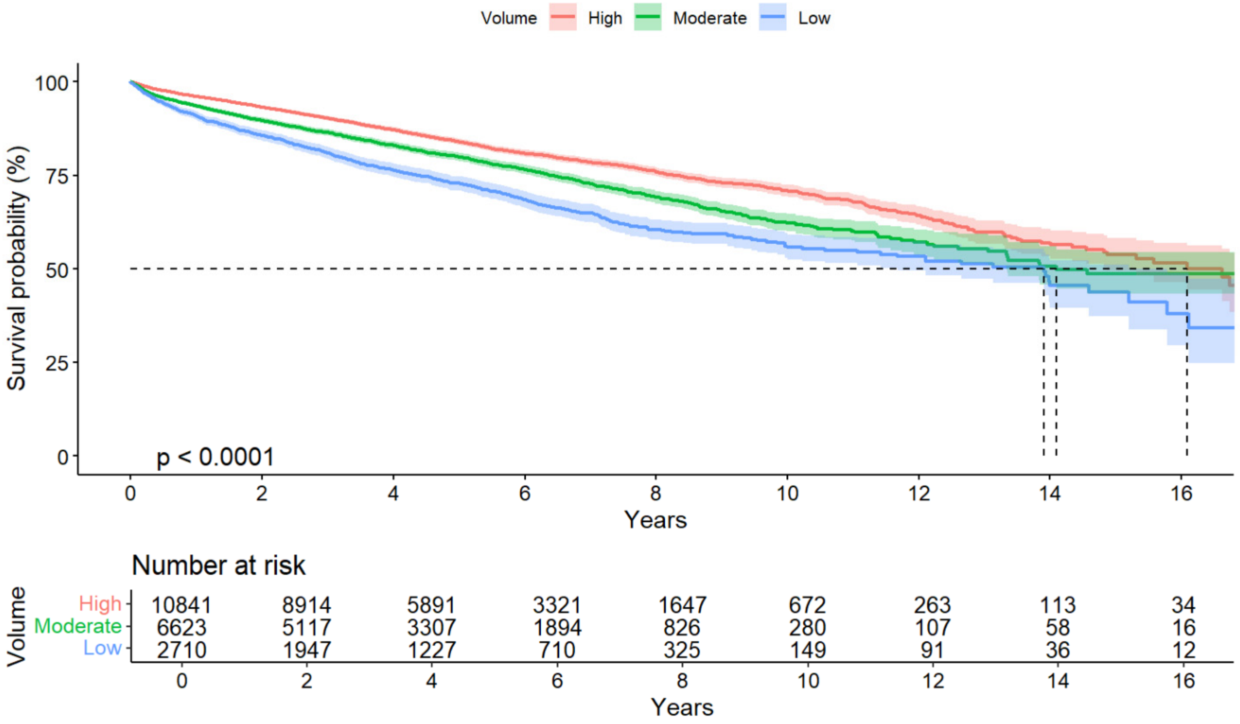


**Figure S1.** Kaplan-Meier Survival Curves by Hospital Volume. *Description*: Survival curves comparing patients treated at high-, moderate-, and low-volume centers. High-volume centers demonstrated superior survival, with a median survival of 16 years, compared to 14 years at moderate- and low-volume centers. *Statistical Tests:* Log-rank test with $p < 0.05$ considered statistically significant and 95 CI.

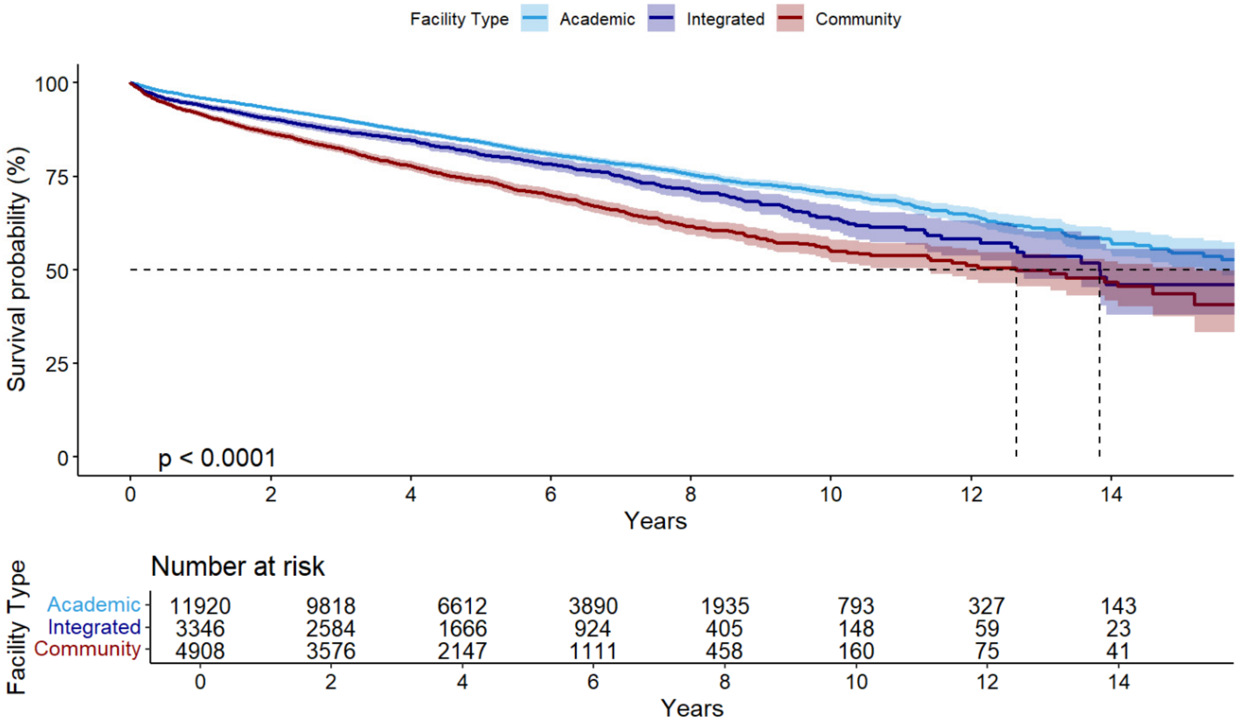


**Figure S2.** Kaplan-Meier Survival Curves by Facility Type. *Description*: Survival curves comparing academic, integrated, and non-academic hospitals. Academic hospitals showed the highest 15-year survival rate, followed by integrated hospitals and community hospitals. *Statistical Tests:* Log-rank test with $p < 0.05$ considered statistically significant and 95 CI.

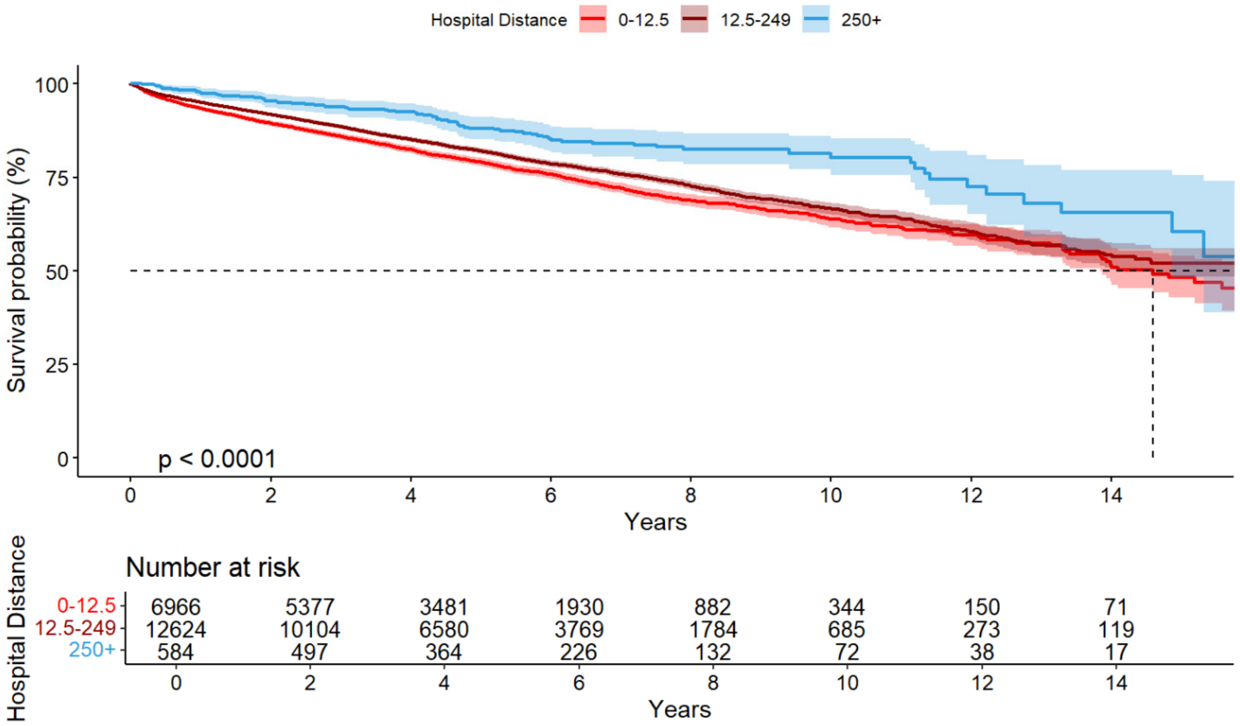


**Figure S3.** Kaplan-Meier Survival Curves by Geographic Distance to Treatment Facility. *Description*: Survival curves comparing patients traveling 0–12.5 miles, 12.5–249 miles, and > 250 miles for care. Patients traveling > 250 miles demonstrated the highest survival rates. *Statistical Tests:* Log-rank test with $p < 0.05$ considered statistically significant and 95 CI.
